# Effect of point mutations on *Herbaspirillum seropedicae* NifA activity

**DOI:** 10.1590/1414-431X20154522

**Published:** 2015-07-10

**Authors:** B. Aquino, A.A. Stefanello, M.A.S. Oliveira, F.O. Pedrosa, E.M. Souza, R.A. Monteiro, L.S. Chubatsu

**Affiliations:** 1 Departamento de Bioquímica e Biologia Molecular, Universidade Federal do Paraná, Curitiba, PR, Brasil

**Keywords:** Biological nitrogen fixation, *Herbaspirillum seropedicae*, NifA

## Abstract

NifA is the transcriptional activator of the *nif* genes in Proteobacteria. It is usually regulated by nitrogen and oxygen, allowing biological nitrogen fixation to occur under appropriate conditions. NifA proteins have a typical three-domain structure, including a regulatory N-terminal GAF domain, which is involved in control by fixed nitrogen and not strictly required for activity, a catalytic AAA+ central domain, which catalyzes open complex formation, and a C-terminal domain involved in DNA-binding. In *Herbaspirillum seropedicae*, a β-proteobacterium capable of colonizing Graminae of agricultural importance, NifA regulation by ammonium involves its N-terminal GAF domain and the signal transduction protein GlnK. When the GAF domain is removed, the protein can still activate *nif* genes transcription; however, ammonium regulation is lost. In this work, we generated eight constructs resulting in point mutations in *H. seropedicae* NifA and analyzed their effect on *nifH* transcription in *Escherichia coli* and *H. seropedicae*. Mutations K22V, T160E, M161V, L172R, and A215D resulted in inactive proteins. Mutations Q216I and S220I produced partially active proteins with activity control similar to wild-type NifA. However, mutation G25E, located in the GAF domain, resulted in an active protein that did not require GlnK for activity and was partially sensitive to ammonium. This suggested that G25E may affect the negative interaction between the N-terminal GAF domain and the catalytic central domain under high ammonium concentrations, thus rendering the protein constitutively active, or that G25E could lead to a conformational change comparable with that when GlnK interacts with the GAF domain.

## Introduction

Biological nitrogen fixation is a process carried out by some prokaryotes that reduces dinitrogen (N_2_) to ammonia (NH_3_) in a reaction catalyzed by the nitrogenase complex. It is highly energy-demanding and is thus controlled at both transcriptional and translational levels (1). Transcription of the *nif* genes, which encode the nitrogenase complex and all gene products necessary to assemble an active enzyme, is controlled by NifA in response to ammonium and oxygen levels. NifA is a σ^54^-dependent transcriptional activator that shows a typical three-domain structure. The N-terminal GAF domain shows the lowest similarity among NifA homologs, and is involved in ammonium control. The central AAA+ domain interacts with the σ^54^-RNA polymerase and possesses ATPase activity, while the C-terminal domain shows a helix-turn-helix motif involved in DNA-binding. Two linkers connect these domains: the Q-linker connects GAF and central domains, and the ID-linker connects the central and C-terminal domains.

NifA proteins are separated into two classes based on their regulation by ammonium and oxygen (1). One class occurs in γ-Proteobacteria and is regulated by the anti-activator NifL, while the second class is observed in α-Proteobacteria, where NifL is absent. Nitrogen regulation by both mechanisms involves a PII-like protein and interaction with either NifL or NifA (2). In contrast, oxygen control differs between these two mechanisms. In γ-Proteobacteria, NifL senses oxygen levels through a flavin moiety (3), whereas in NifL-independent regulation, oxygen control is hypothesized to involve a putative Fe-S cluster associated with a cysteine tetrad located at the end of the central domain and ID-linker (4).

*Herbaspirillum seropedicae* is a nitrogen-fixing β-proteobacterium associated with important agricultural Gramineae, such as rice, wheat, sorghum, and sugarcane (5). Transcriptional control of nitrogen fixation in *H. seropedicae* relies on a NifL-independent NifA system that is controlled by both nitrogen and oxygen levels (6). Recently the regulation of nitrogen fixation in this organism has been reviewed (7).

**Figure 1:**
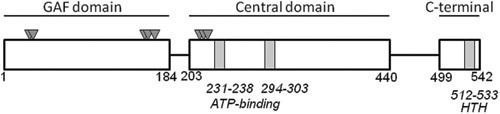
Scheme of Herbaspirillum seropedicae NifA domains. Numbers indicate the amino acid position on the primary structure. The N-terminal GAF domain, central domain, and C-terminal domain are indicated as open rectangles. The ATP-binding motifs, located in the central domain, and the helix-turn-helix (HTH) motif in the C-terminal domain are indicated as gray rectangles, and were predicted using the ScanProsite tool (http://prosite.expasy.org/scanprosite) (29) and Gym2.0 (30), respectively. Point-mutations (K22V, G25E, T160E, M161V, L172R, A215D, Q216I, and S220I) are indicated by gray triangles in the N-terminal GAF and central domains of NifA.

The *H. seropedicae* NifA N-terminal GAF domain comprises the first 184 amino acids (Figure 1), and although it is involved in negative regulation by ammonium, it is not strictly required for activation of *nif* gene transcription (6,8). This N-terminal GAF domain interacts with GlnK in response to the fixed nitrogen concentration (9). The N-terminal GAF domain is linked to the central domain by the 18 amino acids of the Q-linker. The *H. seropedicae* NifA central domain comprises 236 amino acids and contains the catalytic site. It also interacts with the σ^54^ RNA polymerase holoenzyme (4). The central domain is linked to the C-terminal domain by a 58-amino acid region named the ID-linker. A conserved cysteine motif located at the end of the central domain and the ID-linker (positions 414, 426, 446, and 451) is suggested to be involved in the regulation of NifA by O_2_. Mutation of these cysteine residues produces inactive proteins (10). Finally, the last 43 amino acids of the NifA primary sequence form the C-terminal domain, which is responsible for DNA binding (11).

In this work, site-directed mutagenesis was used to determine amino acid residues in *H. seropedicae* NifA that are important for its control.

## Material and Methods

### Reagents

All chemicals were analytical or molecular biology grade and were purchased from Merck (Germany), Sigma (USA), J.T. Baker (Netherlands), or Invitrogen (USA). Restriction enzymes were obtained from Fermentas (Lithuania) or Invitrogen. Oligonucleotides were synthesized by IDT (USA).

### Bacterial strains and growth conditions

*E. coli* strains TOP10 (Invitrogen) and S17.1 (12) were used for cloning and conjugation procedures. *E. coli* were cultured at 37°C in Luria-Bertani broth, terrific broth, super optimal broth (SOB), or SOB with catabolite repression (SOC) broth media (13). *H. seropedicae* strains (Table 1) were grown in NFbHP medium at 30°C (14) with 37 mM malate and 20 mM NH_4_Cl or 0.5 mM glutamate.

**Table:**
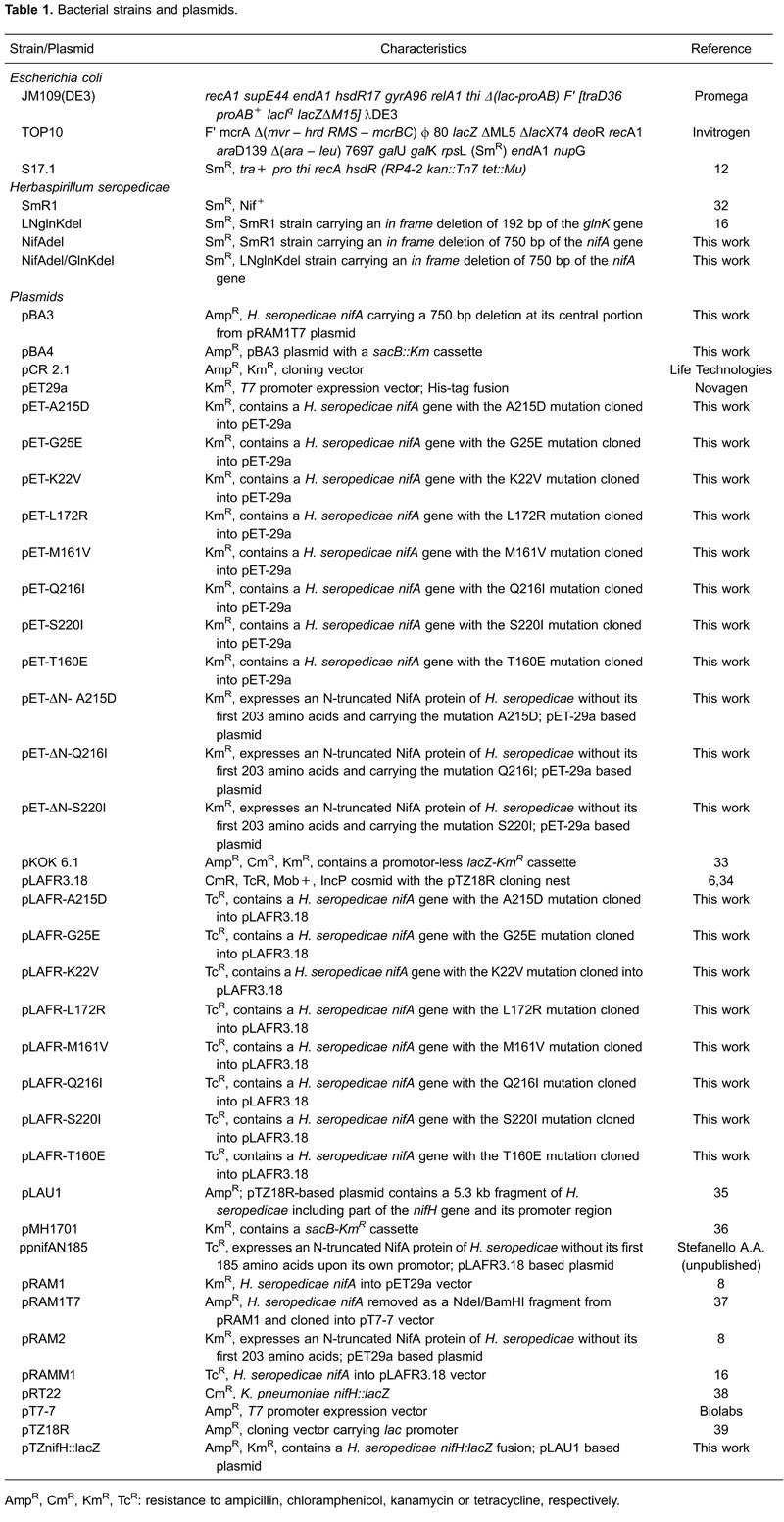


### Site-directed mutagenesis

Point mutations were introduced into the *H. seropedicae nifA* gene using mutagenic primers (Supplementary Table S1) as described previously (15). The mutated genes were then cloned into pET-29a, using NdeI and BamHI restriction sites, or into pLAFR3.18 (XbaI/HindIII restriction sites) for activity analyses in *E. coli* or *H. seropedicae*, respectively. These plasmids are listed in Table 1.

### Construction of *H. seropedicae* mutants

The *nifA* gene, cloned into pRAM1T7 as an NdeI/BamHI fragment, was digested with *Eco*RI to remove 750 bp from the central region of *nifA*. Following re-ligation, the resulting plasmid (pBA3) was digested with *Bam*HI, and a *sacB::Km* cassette, obtained as a BamHI fragment from pMH1701, was introduced into pBA3, producing pBA4. This plasmid was then introduced into *H. seropedicae* SmR1 (wild type) and LNglnKdel, a *glnK* mutant (16), by electroporation (10 kV/cm, 4 kΩ, 330 μF, using a Gibco Cell-Porator, USA). Transformed cells were first selected by growth in NFbHP medium with 1 mg/mL kanamycin, and then by survival in NFbHP medium with 15% (w/v) sucrose to obtain mutants with a second recombination. The *nifA* mutation was confirmed by DNA amplification using 1U Taq DNA Polymerase (Fermentas) in Taq buffer with (NH_4_)_2_SO_4_, 3 mM MgCl_2_, 0.8 mM dNTP and 0.4 μM of primers HSNifA1 and HSNifA2a (Supplementary Table S1) and the following parameters: one step for 5 min at 95^o^C and 30 cycles of 30 s at 95^o^C, 30 s at 45^o^C and 2 min at 72^o^C. These strains were named NifAdel and NifAdel/GlnKdel, respectively.

*H. seropedicae* mutants carrying a chromosomal *nifH::lacZ* fusion were generated by introducing the pTZnifH::lacZ plasmid by electroporation, followed by selection for kanamycin resistance. The pTZnifH::lacZ plasmid was constructed by cloning a *lacZ::Km* cassette at the BamHI site located downstream from the 5.3-kb fragment containing part of *H. seropedicae nifH* and its promoter region in the pLAU1 plasmid. The *lacZ::Km* cassette was obtained as a BamHI fragment from the pKOK6.1 plasmid.

### Protein analyses

β-galactosidase activity was determined as described previously (17). *E. coli* strain JM109(DE3), carrying plasmids pRT22 (*Klebsiella pneumoniae nifH::lacZ*) and pET-29a with different *nifA* mutations, was analyzed as described previously (9). The β-galactosidase activity in *H. seropedicae* was determined as described previously (16). Nitrogenase activity was determined using cells grown in semi-solid NFbHP medium containing glutamate (0.5 mM). Protein concentration was determined using the Bradford method (18) with bovine serum albumin as a standard.

## Results

In this work, we generated eight constructs that introduced NifA point mutations (K22V, G25E, T160E, M161V, L172R, A215D, Q216I, and S220I) and analyzed their effects on transcriptional activation activity. Four mutations were based on described NifA mutations from *Rhodospirillum rubrum* (19) and *Sinorhizobium meliloti* (20), while the other four amino acids were chosen from among conserved residues (Supplementary Table S2). Residues were selected for mutagenesis by aligning NifA proteins from *K. pneumoniae*, *Azoarcus* sp., and *Azotobacter vinelandii*, which are regulated by NifL, and from *H. seropedicae*, *R. rubrum*, *S. meliloti, Rhodobacter capsulatus, Bradyrhizobium japonicum,* and *Azospirillum brasilense*, which are regulated in a NifL-independent manner (Supplementary Table S2). The mutations were located in the N-terminal GAF domain (K22V, G25E, T160E, M161V, and L172R) and the central domain (A215D, Q216I, and S220I) of *H. seropedicae* NifA (Figure 1). The secondary structure of each mutant protein was predicted using the Psipred tool (21), which indicated no major differences in secondary structure between the mutants and wild-type NifA (data not shown).

**Figure 2:**
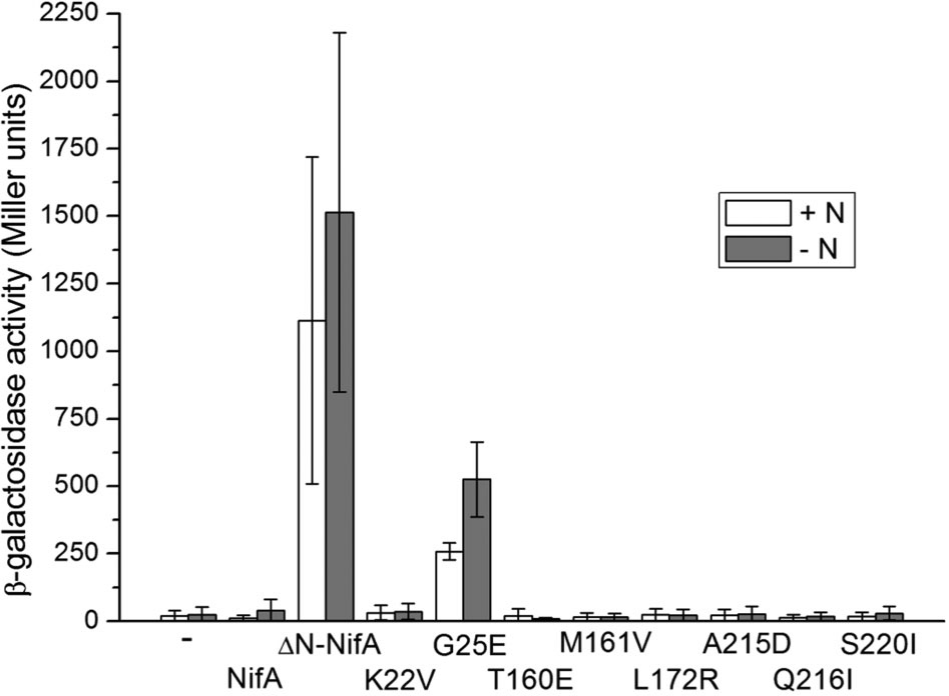
Transcriptional activity of NifA variant proteins in *Escherichia coli* JM109 (DE3) carrying pRT22 (*nifH::lacZ*). (-) indicates cells carrying pET-29a. Full-length NifA was expressed from pRAM1. ΔN-NifA indicates an N-truncated form of NifA expressed from pRAM2. Full-length NifA mutants, as indicated, were expressed from pET-29a-based plasmids. β-galactosidase expression experiments were performed in NFDM medium (31) with 20 mM of ammonium chloride (+N) or 0.2% casamino acids (-N), in the absence of O_2_. Data are reported as the mean±SD of 3 independent assays. β-galactosidase activity is reported as Miller units.

The ability of the *H. seropedicae* NifA mutants to activate *nif* promoters was determined in *E. coli* JM109(DE3) carrying plasmid pRT22 (*K. pneumoniae nifH::lacZ* fusion) (Figure 2). Full-length NifA, expressed from plasmid pRAM1, showed no β-galactosidase activity, consistent with previous descriptions, mainly because of lower expression of endogenous *E. coli* PII, which is necessary to relieve the negative control of the N-terminal GAF domain on the catalytic domain of NifA (7). In contrast, the N-terminal truncated NifA protein (ΔN-NifA) expressed from pRAM2 was fully functional in *E. coli* regardless of the ammonium concentration (22). These results confirmed the regulatory role of the N-terminal GAF domain on *H. seropedicae* NifA that has been described previously: in the presence of ammonium or the absence of PII, the N-terminal GAF inhibits NifA transcriptional activity (6,23). The constructed NifA point mutants were analyzed under the same conditions and showed no activity, except for NifA G25E, which partially activated *nifH* transcription in *E. coli*. G25E also appeared to retain some nitrogen control, as activation of transcription was higher under low ammonium concentrations. This result indicated that the G25E substitution affected the need for PII for NifA activity in *H. seropedicae*.

**Figure 3:**
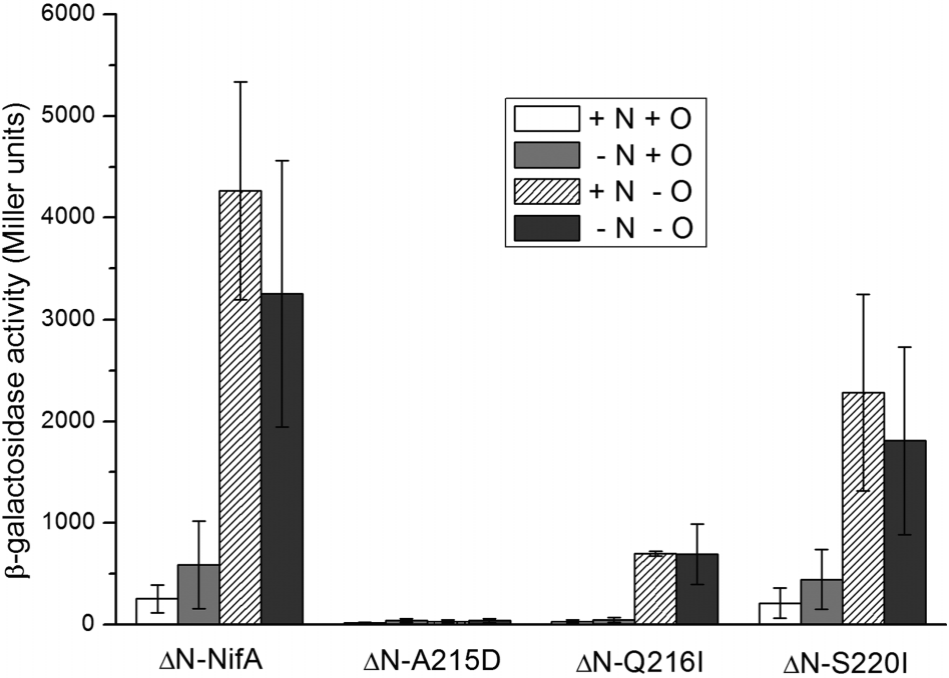
Transcriptional activity of ΔN-NifA mutant proteins in *Escherichia coli* JM109 (DE3) carrying pRT22 (*nifH::lacZ*). ΔN-NifA indicates NifA lacking 203 amino acid residues at the N-terminal GAF domain. ΔN-215D, ΔN-Q216I, and ΔN-S220I indicate the N-truncated forms of NifA mutants. β-galactosidase expression experiments were performed in NFDM medium supplemented with 20 mM ammonium chloride (+N) or 0.2% casamino acids (-N) in the presence (+O) or absence (-O) of O_2_. Data are reported as the mean±SD of 3 independent assays. β-galactosidase activity is reported as Miller units.

Considering that A215D, Q216I, and S220I are located in the central domain of NifA, and that the full-length protein is inactive in *E. coli* (6) (Figure 2), these three mutations were also tested using an N-truncated form (GAF truncated protein) (Figure 3). The removal of the first 203 amino acids of NifA yields an active protein in *E. coli* (8), as shown using protein expressed from plasmid pRAM2. The N-truncated protein (ΔN-NifA) was active regardless of the nitrogen level, but only under low O_2_, reinforcing its sensitivity toward O_2_. The N-truncated ΔN-Q216I and ΔN-S220I mutants showed lower β-galactosidase activity than that expressed by pRAM2, indicating that these mutations negatively affect transcriptional activity, while retaining O_2_ responsiveness. In contrast, ΔN-A215D was inactive under all tested conditions, suggesting that a negatively-charged amino acid at position 215 affects the catalytic activity of the protein. These proteins were expressed under all conditions tested, as determined by gel electrophoresis (data not shown). The three disrupted amino acids are close to the ATP-binding site, which is located at positions 231-238. In contrast to *H. seropedicae*, a NifA strain with a mutation in this region (M217I) in *S. meliloti* was oxygen tolerant (20).

The point mutations were also analyzed in an *H. seropedicae* background. Assuming that a functional NifA variant leads to *nif* gene transcription, nitrogenase activity can be determined. However, for these assays it was necessary to construct two *H. seropedicae* mutant strains: a *nifA* mutant strain and a double mutant *nifA/glnK*, both of which were obtained by partial gene deletion. These strains were named NifAdel and NifAdel/GlnKdel, respectively. The *nifA/glnK* double mutant allowed detection of a NifA mutant that does not require GlnK for activity, as this PII protein is responsible for relieving the nitrogen-regulated negative control of NifA (16). These *H. seropedicae* mutant strains showed no nitrogenase activity (acetylene reduction method; Table 2) (24,25). However, the nitrogenase activity was restored in the pRAMM1-carrying NifAdel strain, which expresses the full-length NifA, and in the NifAdel/GlnKdel strain carrying ppnifAN185, which expressed an N-truncated form of NifA (Table 2).

**Table:**
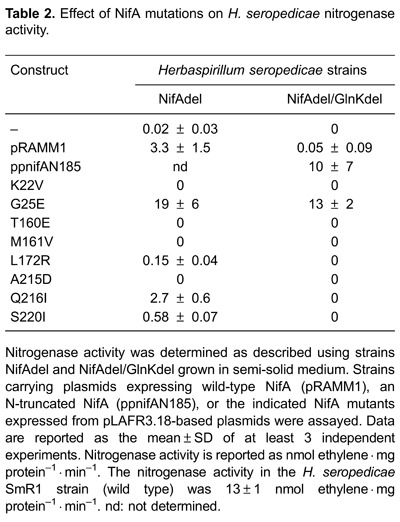


To analyze the effect of NifA mutations on nitrogenase activity, each construct was cloned into the pLAFR3.18 vector, which is stable in *H. seropedicae,* and transformed into both the NifAdel and NifAdel/GlnKdel strains. Assays performed with NifAdel showed that the G25E mutant was fully active, Q216I was partially active, and the other mutants showed no significant nitrogenase activity (Table 2). However, NifA levels similar to those of the wild type were expressed from pRAMM1, while the Q216I mutant did not show any nitrogenase activity in the absence of GlnK (assay in NifAdel/GlnKdel strain). In contrast, the G25E mutant demonstrated nitrogenase activity, which implied that G25E was active and does not require GlnK for activity.

**Figure 4:**
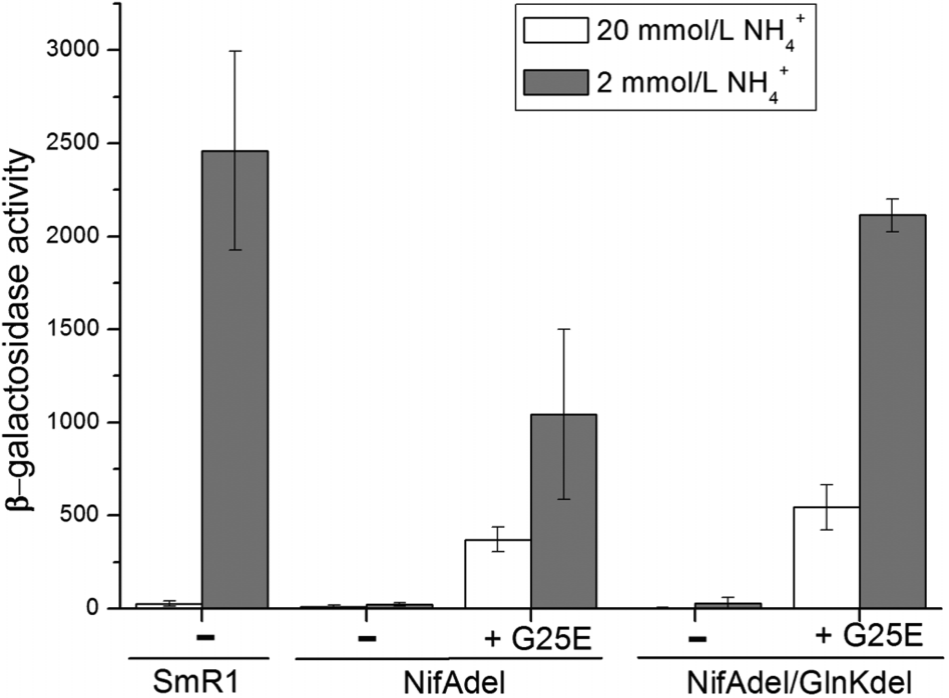
Transcriptional activity of indicated *Herbaspirillum seropedicae* strains carrying a chromosomal *nifH:lacZ* fusion. +G25E indicates cells carrying a pLAFR3.18-based plasmid expressing the G25E NifA mutant; (-) indicates absence of plasmid. Cells were grown in NFbHP medium supplemented with 10 mM NH_4_Cl under aerobic conditions at 30°C. Cells were then centrifuged (1700 *g* for 2 min), resuspended in NFbHP (nitrogen-free) medium, and de-repressed for 7 h under 1.5% oxygen. β-galactosidase was determined as described. Data are reported as the mean±SD of at least 3 independent experiments. β-galactosidase activity is reported as Miller units.

The G25E mutation was also tested in the NifAdel and NifAdel/GlnKdel strains carrying a *nifH::lacZ* chromosomal fusion, which allowed assessment of transcriptional NifA activity in the presence of high ammonium concentrations (Figure 4). The wild-type *H. seropedicae* strain (SmR1) carrying the *nifH::lacZ* fusion only showed β-galactosidase activity at low ammonium concentrations. Conversely, the G25E mutant showed *nifH::lacZ* transcription in both the NifAdel and NifAdel/GlnKdel strains, regardless of ammonium concentration. However, comparison of β-galactosidase activity at both low and high ammonium concentrations indicated that the G25E mutant protein was partially regulated by ammonium, as transcriptional activity was higher at low ammonium concentrations. This result suggested that the G25E mutant did not depend on GlnK for activity, but could still detect ammonium concentration.

## Discussion

*H. seropedicae* NifA is regulated by both ammonium and oxygen (6). The effect of O_2_ on the NifA protein is related to a putative Fe-S cluster involving four cysteine residues located at the end of the central domain and the ID-linker. These conserved cysteine residues are found in all NifA proteins that are directly sensitive to oxygen, but absent in NifA proteins that depend on NifL for oxygen control (4). In *H. seropedicae*, mutation of the conserved cysteine residues rendered inactive proteins (10). Conversely, Krey et al. (20) obtained a *S. meliloti* NifA mutant (M217I) that was active even under high O_2_ levels. Using sequence alignment, we determined the corresponding amino acid residue in *H. seropedicae* to be serine 220. The NifA S220I mutation resulted in lower activity in *H. seropedicae* (Table 2) and partial activity in *E. coli* (Figure 3), but retained sensitivity toward O_2_, indicating a difference in behavior compared with *S. meliloti* NifA M217I.

Two further amino acid residues close to S220 in *H. seropedicae* NifA were also mutated and analyzed. The A215D mutation was benign in both *H. seropedicae* and *E. coli*, even if an N-truncated form was used. However, the N-truncated protein form carrying the Q216I mutation showed transcriptional activity dependent on O_2_. This mutant Q216I *H. seropedicae* strain also produced an active nitrogenase complex dependent on the GlnK protein, similar to the wild type. These results indicate that Q216I and S220I retained regulatory activities similar to the wild type, although with lower activity. Conversely, because no transcription from strains carrying the A215D mutation was observed under any of the conditions tested, the alanine residue at position 215 is likely to be essential for activity. Alternatively, a negative charge at position 215 may be more deleterious for *H. seropedicae* NifA compared with the previous substitutions.

Mutations M161V and L172R in *H. seropedicae* NifA correspond to mutations M173V and L184R described previously in *R. rubrum* (19). Analysis carried out using a yeast two-hybrid system showed that *Rr*M173V produced a protein with stronger GlnB interaction, whereas the *Rr*L184R mutant did not require GlnB for activity. In *R. rubrum*, GlnB is the PII protein responsible for controlling NifA activity (26). The *H. seropedicae* NifA M161V mutant was inactive in all conditions tested, while the L172R mutant showed very low nitrogenase activity, indicating that these amino acids are important for the overall NifA activity.

*H. seropedicae* differs from *R. rubrum* in that nitrogen regulation depends on GlnK (16). Among the eight *H. seropedicae* NifA mutations investigated in this work, the G25E mutation rendered an active NifA protein that did not require GlnK. Mutation G25E in *H. seropedicae* corresponds to G36E in *R. rubrum*, which also produced a partial active NifA independent of GlnB (19). The G25E mutation may affect the negative regulatory interaction between the N-terminal GAF domain and the catalytic central domain under high ammonium concentrations, resulting in a constitutively active protein. Furthermore, the glutamate residue at position 25 could lead to a conformational change comparable with that produced when GlnK interacts with the N-terminal GAF domain.

The G25E mutant was also analyzed using a *nifH:lacZ* chromosomal fusion in *H. seropedicae* (Figure 4). This allowed us to determine the NifA transcriptional activity even in the presence of high ammonium concentrations, a condition where nitrogenase activity is not observed (16). The assay showed that the G25E mutant is active in the absence of GlnK, as observed by nitrogenase activity, but also showed partial regulation by fixed nitrogen, with higher transcriptional activity under low ammonium concentrations than in the presence of high ammonium concentrations (Figure 4). The partial regulation by fixed nitrogen was also observed in assays performed in *E. coli* (Figure 2).

The observed ammonium regulation could be related to the GAF domain, which has been shown to bind small molecules such as cyclic nucleotides and 2-oxoglutarate (27). In *A. vinelandii*, formation of the NifL-NifA complex is prevented by the binding of 2-oxoglutarate to the NifA GAF domain (28). Although it has not been confirmed that the N-terminal GAF domain of *H. seropedicae* NifA binds small molecules, the possibility that a small molecule such as 2-oxoglutarate may interact with the protein, signaling a cellular deficit of fixed nitrogen, cannot be ruled out.

## Supplementary material

 Click here to view [pdf]
